# Is there a link between diet and painful temporomandibular disorders? A cross-sectional study

**DOI:** 10.1186/s12903-025-06835-0

**Published:** 2025-09-17

**Authors:** Camila Cury Marques, Pedro Miguel Teixeira Carvas Cebola, Idoya Orradre Burusco, Maria García González, Miguel de Pedro, Nikolaos Christidis, Malin Ernberg, Giancarlo De la Torre Canales

**Affiliations:** 1 Egas Moniz Center for Interdisciplinary Research (CiiEM), Egas Moniz School of Health and Science, Almada, Portugal; 2 Orofacial Pain Unit and Sleep Medicine Center, CUF Tejo Hospital, Lisboa, Portugal; 3 Orofacial Pain, Oral Pathology and Dental Sleep Medicine Research Group, Department of Postgraduate Clinical Dentistry, Faculty of Biomedical Sciences, European University of Madrid, Madrid, Spain; 4 Department of Dentistry, Ingá University Center, Uningá, Paraná Brazil; 5 Division of Oral Rehabilitation, Department of Dental Medicine, Karolinska Institutet, Huddinge, Sweden

**Keywords:** Temporomandibular disorders, Diet, Nutrition

## Abstract

**Background:**

Nutrition and diet have emerged as potentially modifiable factors in the management of chronic pain, however, there is still limited evidence regarding the direct relationship between diet, nutrition, and Temporomandibular disorders (TMD). The aim of this cross-sectional study was to explore the relationship between dietary patterns and painful TMD, with a particular focus on dietary inflammatory potential, adherence to the Mediterranean diet, and psychosocial predictors.

**Methods:**

Ninety-two participants (45 TMD patients and 47 controls) aged 20–50 were recruited in Portugal and Spain. TMD diagnosis was based on the Diagnostic Criteria for TMD (DC/TMD). Psychosocial status was assessed using PHQ-9 (depression), PHQ-15 (somatic symptoms), and the Oral Behavior Checklist (OBC). Dietary data were collected through a 24-hour recall and assessed using the Healthy Eating Index (HEI), Dietary Inflammatory Index (DII), and Mediterranean Diet Adherence Screener (MEDAS). Pressure pain thresholds (PPT) were recorded at the TMJ and masticatory muscles. Data was analyzed using independent t-tests, Mann-Whitney U, and OPLS-DA multivariate modeling.

**Results:**

TMD patients showed significantly higher scores for somatic symptoms, depressive symptoms, and maladaptive oral behaviors. While no significant differences were found for DII or MEDAS scores between groups, TMD patients had significantly lower HEI scores and PPTs values in the masseter, temporalis, and TMJ regions. OPLS-DA identified oral behaviors, somatic symptoms, and lower HEI as the strongest predictors distinguishing TMD patients from controls.

**Conclusions:**

Painful TMD is associated with higher psychosocial distress and poorer dietary quality. These findings underscore the need to include dietary assessment in the clinical evaluation and management of TMD.

**Supplementary Information:**

The online version contains supplementary material available at 10.1186/s12903-025-06835-0.

## Background

Temporomandibular disorders (TMD) encompass a diverse range of musculoskeletal conditions that primarily impact the temporomandibular joints (TMJ), masticatory muscles, and associated tissues [1]. These disorders are recognized as the leading cause of chronic orofacial pain that is not attributable to dental issues [2]. Epidemiological data indicate that approximately 34% of the adult population worldwide is affected by TMD, with women of reproductive age being disproportionately affected compared to men [2, 3]. Additionally, findings from the Orofacial Pain: Prospective Evaluation and Risk Assessment (OPPERA) prospective cohort study suggest that the annual incidence rate of painful TMD among adults in the United States is 3.9% [2].

The precise etiology of TMD remains unclear; however, it is recognized as being biopsychosocial in nature and multifactorial, influenced by psychosocial factors, autonomic responses, genetic predispositions, and states of pain amplification [4, 5]. Consequently, TMD is frequently associated with significant pain and possesses the potential to develop into a chronic condition, adversely affecting an individual’s quality of life [6–8]. Among those with TMD, the experience of pain can lead to a decline in masticatory performance, which significantly impacts dietary intake and eating behaviors [5, 9]. TMD individuals with chewing difficulties or those who fear exacerbating symptoms through chewing often exhibit a tendency to prefer softer food options, which in turn, could lead to subclinical nutritional deficiencies [10]. Furthermore, individuals who limit their food intake due to diminished masticatory performance are frequently classified within the initial two stages of protein-energy malnutrition [11].

In recent years, nutrition has emerged as a potentially modifiable factor in the management of chronic pain, given that dietary habits are believed to affect pain processing, particularly through their impact on low-grade inflammation [12]. Diets characterized by high consumption of ultra-processed foods, trans fats, and refined sugars have been associated with inflammatory responses, which may exacerbate chronic pain conditions. Conversely, anti-inflammatory dietary patterns, such as the Mediterranean diet, have been linked to more favorable pain outcomes [13]. Specifically, deficiencies in omega-3 fatty acids (essential for optimal function of the central nervous system (CNS)), vitamin D, polyamines, and magnesium have been associated with increased inflammatory pain [12]musculoskeletal pain and pain sensitization, respectively [12, 14].

Regarding TMD, a study reported that the severity of myofascial TMD pain is associated with dietary changes, with patients experiencing difficulties in chewing even soft foods reporting reduced intake of essential nutrients, including calcium, iron, and fiber [15, 16]. Nonetheless, when compared to a general population sample, no significant differences were observed in nutrient intake indicators [16]. TMD management may also adversely affect dietary habits, as participants often adhere to soft food recommendations that have not been adequately evaluated for their effects on TMD symptoms or their influence on nutritional status [17]. Conversely, dietary modifications like adopting gluten-free diets or implementing targeted micronutrient supplementation have been shown to reduce myofascial TMD pain [18, 19]. There is still limited evidence regarding the direct relationship between diet, nutrition, and TMD. The present study aimed to investigate the impact of various dietary patterns on TMD and its influence on clinical outcomes.

## Methods

This study received approval from the Research Ethics Committee of Egas Moniz School of Health and Sciences (PT-211/24), CUF TEJO Hospital (4/2024/537) and European University of Madrid (2024 − 818). Included participants provided a written and signed inform consent to participate in the trial. The study adhered to the Helsinki Declaration, and was performed at the Egas Moniz Dental Clinic, CUF TEJO Hospital and European University Dental Clinic from September 2024 to March 20,245. The reporting of data followed the Strengthening the Reporting of Observational Studies in Epidemiology (STROBE) guideline [20].

### Participants

The sample was obtained from Portuguese and Spanish individuals, seeking regular dental (healthy control group) or TMD treatment at Egas Moniz Dental Clinic, CUF Tejo Hospital and European University of Madrid.

Inclusion criteria were volunteers of both sexes, aged between 20 and 50 years, with or without diagnosis of TMD, according to the Portuguese and Spanish version of the Diagnostic Criteria for Temporomandibular Disorders (DC/TMD) – found at the official website https://inform-iadr.com/ – assessed by four dentists trained to use the DC/TMD and specialized in orofacial pain (8 years). The exclusion criteria were patients with missing molars or proper prosthodontics treatment, who had received previous treatments for TMD, undergoing orthodontic treatment, with pain of dental and neuropathic origin, traumas to the face or neck, and with rheumatic and/or psychiatric diseases.

The sample size calculation based on a previous study [21] was performed using the variable Healthy Eating Index, with the G*Power 3.1.9.2 software (Düsseldorf, Germany). The following parameters were considered: a power of 0.95, a significance level of 0.05, an effect size of 0.5 with a SD1 of 0.63. The calculation indicated the need for at least 42 participants per group. The final sample was composed of 92 participants, divided into two groups: 45 in the TMD group and 47 healthy controls.

### Study protocol

Participants were assessed once in this study. During this visit, they were screened based on the study’s inclusion and exclusion criteria. Volunteers were first assessed through the TMD Pain Screener [22]included in the DC/TMD and only those with at least one positive answer were evaluated with the complete DC/TMD protocol [23]. Participants with all negative answers were allocated in the control group. Then, study protocol and methods were explained to the participants before the study started.

### Outcomes

#### Pain intensity and sensitivity

The Characteristic Pain Intensity (CPI) was assessed by numeric rating scales included in questions #2 to 4# of the Graded Chronic Pain Scale (GCPS) [24]. The CPI was defined as the mean value of these three questions (current, average, and worst pain intensity) multiplied by 10 [23, 25].

For Pressure Pain Threshold (PPT) measurements, a digital algometer (Kratos DDK-20) with a flat circular tip of 1 cm², was used. A constant and increasing pressure of approximately 0.5 kg/cm²/sec was applied to the assessed structures. The assessments were performed in the most painful site in the TMD group and in the dominant site in the control group. Participants were instructed to press a button connected to the device itself to indicate the moment at which the sensation of pressure transformed into a painful stimulus [26, 27]. The arithmetic mean of the three measurements performed at the lateral pole of the TMJ, temporalis and masseter muscles were considered the final PPT value for statistical analysis.

#### Psychological status measurements

To assess the psychological aspects related to TMD, the following validated self-report questionnaires were applied. *Patient Health Questionnaire (PHQ-9)* [28] used to screen depression and contains nine questions, each scored 0–3 based on frequency, with a total score ranging from (0–27). Scoring considers mild (5–9), moderate (10–14), moderate-severe (15–19) and severe depression (20+). *The Somatic Symptom scale (PHQ-15)* [29] evaluates the presence and severity of nonspecific somatic (physical) symptoms/somatization. A 0–3 scored per question is given according to frequency, with a total score of 0–30 and scores of ≥ 5, ≥10 and ≥ 15 points specify mild, moderate, and high somatic symptoms/somatization, respectively.

#### Oral behavior checklist (OBC)

The OBC consists of 21 questions regarding oral behaviors. These items encompass self-reported awake bruxism over the last 30 days. For each item is used a scale from 0 referring “none of the time” to 4 denoting “all of the time”. Total scores represent normal (0–16), moderate (17–24) and severe (25–62) oral behaviors [30].

#### Diet assessments

##### Food consumption

Food consumption was estimated through one 24-hour dietary recall (R24h), in which participants reported all food and beverages consumed considering household measures in the previous 24 h. The R24h is a validated instrument widely used in research to assess patients’ dietary profiles (13,14) and was used to assess the Healthy Eating Index and the Dietary Inflammatory Index. Initially, three R24h was considered for this study, however none of the patients completed in a proper manner more than one R24h.

##### Calculation of the healthy eating index and the Dietary Inflammatory Index (DII)

Food consumption was used to determine the Healthy Eating Index (HEI) and the Dietary Inflammatory Index (DII). The HEI index is a 100-point analytical tool used to measure adherence to dietary guidelines and recommendations, determining compliance with the Food Pyramid recommendations for the five main food groups: grains, vegetables, fruits, milk, and meat. It assesses the consumption of total and saturated fat, sodium, cholesterol, and dietary variety of the individual. The scores of the 10 components can range from 0 to 10, with the lowest value indicating no consumption. Scores > 80 points represent a good diet; 51–80 points denotes a fair diet; and < 50 points represent a poor diet [31].

To calculate DII scores, the method previously proposed by Shivappa et al., 2014 was used. This method was based on the analysis and review of 1,943 studies, evaluating foods and nutrients and their effects on six inflammatory markers: Interleukin (IL) 1-beta (IL-1β), IL-4, IL-6, IL-10, tumor necrosis factor-alpha (TNF-α) and C-Reactive Protein (CRP), was used. In the study, the authors categorized 45 dietary components as pro-inflammatory and anti-inflammatory [32]. In the present study, the following 25 dietary components were analyzed: Energy (kcal), proteins, carbohydrates, total fat, cholesterol, saturated fatty acids, monounsaturated fatty acids, polyunsaturated fatty acids, fibers, additional sugar, vitamin B6, vitamin B12, vitamin B9 vitamin A, vitamin C, vitamin D, vitamin E, iron, magnesium, calcium, zinc, alcohol, tea, caffeine and curcuma. Dietary intake of the DII components of the participants was compared to the standard global as a Z-score [32]. Then, this value was converted to a centered percentile score. To achieve a symmetrical distribution with values centered on 0 (null) and bounded between − 1 (maximally anti-inflammatory) and + 1 (maximally pro-inflammatory), each percentile score was doubled and then ‘1’ was subtracted. The centered percentile values were then multiplied by the overall pro- and anti-inflammatory effect score for each dietary component. Finally, results were summed up. Negative scores are more anti-inflammatory, and more positive scores are pro-inflammatory. For the overall total, higher scores indicate a more pro-inflammatory diet and lower scores represent a more anti-inflammatory diet.

##### Mediterranean Diet Adherence Screener (MEDAS)

MEDAS is a 14-item screener, which consists of 12 questions on food consumption frequency and 2 questions on food intake habits characteristic of the Mediterranean diet. Each question is scored with a 0 or 1. Then, the Portuguese and Spanish versions of the MEDAS were used to evaluate the frequency of consumption of various foods characteristics of the Mediterranean diet, such us fruits, vegetables, nuts, olive oil, fish, legumes, lean meats, and whole grains [33].

### Statistical analysis

All data were analyzed using SPSS Statistics 25.0 software (IBM, New York, USA). Initially, data was tabulated into a spreadsheet and organized. The Shapiro-Wilk test assessed data distribution. For age, BMI and PPT, comparisons between groups were made using the independent T-test. As psychosocial variables (PHQ-9, PHQ-15, OBC,) are composed of scores obtained from individual ordinal scales, the sum scores cannot be regarded as continuous, therefore for these variables and for HEI, DII and MEDAS the U Mann-Whitney test was used for groups comparisons. The chi-squared test analyzed gender and country frequencies. A 5% probability level was considered as significant in all tests.

Multivariate statistics were used to identify outcomes that separate the groups using SIMCA-P + V.17.0 (Sartorius Stedim Biotech, Umeå, Sweden) [34]. Principal component analysis (PCA) was first used to investigate the correlation for the investigated variables and to detect moderate or strong outliers using Hotelling´s T2 and DMod. Three outliers were detected and excluded from the analysis. Then, orthogonal partial least squares discriminant analysis (OPLS-DA) was used to regress group membership (multivariate correlations between variables and group). R2 describes the goodness of the fit, whereas Q2 describes the goodness of prediction. R2 should not be considerably higher than Q2, if substantially higher (> 0.3) the robustness of the model is poor [35]. To validate the model, CV-ANOVA was used to test the significance of the model. If the CV-ANOVA had a *p* value < 0.05, the OPLS-DA model was considered significant. The variable influence on projection (VIP) value indicates the relevance of each X-variable pooled over all dimensions and the Y-variables indicate the group of variables that best explain Y. P (corr) was used to note the direction of the relationship (positive or negative). VIP > 1.0 and an absolute p(corr) > 0.4 was considered significant.

## Results

### Sample characteristics

Overall, 130 patients were screened; 38 (29.2%) failed the stablished criteria. The sample was composed of 92 patients, 45 individuals in the TMD group (40.3 ± 14.4) and 47 healthy controls (37.2 ± 10.1). No significant differences in age, gender and body mass index (BMI) were observed between groups (*p* > 0.05). The TMD group presented the following frequency of diagnoses: myalgia, 44.1%; arthralgia, 2.2%; disk displacement with reduction, 48.8%; disk displacement without reduction, 2.2% and multiple diagnoses 37%. The median (min-max) pain intensity of the TMD group was 57 (23–90) and 72% of the subjects in this group presented a pain duration ≥ 3 months (Table 1).

**Table 1:** Comparisons of demographic variables between TMD patients and controls (mean ± sd and n (%))

	Groups			
	TMD (*n* = 45)	Controls (*n* = 47)		*p*
Age	40.3 ± 14.4	37.2 ± 10.1		0.24
*Gender*				
Women	37 (82)	32 (68)		0.11
Men	8 (18)	15 (32)		
*Country*				
Portugal	25 (56)	27 (57)		0.21
Spain	20 (44)	20 (43)		
*BMI*	24.1 ± 2.6	24.4 ± 4.6		0.42
*TMD painful diagnosis*				
Myalgia	56 (25)	--		
Arthralgia	2.2 (1)	--		--
DDWR	48.8 (22)	--		
DDWoR	2.2 (1)	--		
Myalgia + Arthralgia	44 (19)	--		

*BMI* Body mass index, *DDWR* Disk displacement with reduction, *DDWoR* Disk displacement without reduction *TMD* Temporomandibular disorders

*p<0.05

### Psychosocial, nutrition and PPT variables

Scores for all psychosocial and nutrition questionnaires and PPT are shown in Table 2. Inter-group comparisons showed higher values for the TMD group compared with controls for PHQ-9, PHQ15 and OBC (*p* = 0.001). Regarding nutrition assessments, the TMD group presented lower values for HEI (*p* = 0.001), however no significant differences were found between groups for DII and MEDAS (*p* > 0.05). Additionally, a significant less consumption of Vitamin C (*p* = 0.027) and B12 (*p* = 0.003), and garlic (*p* = 0.007) were found in the TMD group. On the other hand, the TMD group presented a higher intake of carbohydrates (*p* = 0.007) (S1). Furthermore, the TMD group presented lower PPTs values compared with controls for temporalis (*p* = 0.03) and masseter (*p* = 0.04) muscles and TMJ (*p* = 0.01).

**Table 2:** Comparisons of assessed variables between TMD patients and controls (Mean ± sd and max (min-max)

	Groups			
	TMD (*n* = 45)	Controls (*n* = 47)		*p*
*Psychosocial Variables*				
PHQ-9	5 (0–23)	2 (0–12)		0.001^*^
PHQ-15	8 (0–19)	3 (0–12)		0.001^*^
OBC	25 (14–53)	11 (0–19)		0.001^*^
*Nutricional Variables*				
HEI	68.03 (34.4–89.9)	87.02 (55.0–90.0)		0.001^*^
DII	−0.02 (−2.2-2.5)	−0.57 (−3.1-1.3)		0.05
MEDAS	7.0 (2.0–11.0)	7.0 (0–19.0)		0.47
*PPT*				
Temporalis	1.9 ± 1.3	2.7 ± 1.9		0.03^*^
TMJ	1.3 ± 1.0	2.1 ± 1,2		0.01^*^
Masseter	1.6 ± 1.3	2.8 ± 1,4		0.04^*^

*HEI* Healthy Eating Index, *DII* Dietary Inflammatory Index, *MEDAS* Mediterranean Diet Adherence Screener, *OBC* Oral Behaviour Checklist, *PHQ-15* Patient Health Questionnaire-15 (somatic symptoms), *HEI* Healthy Eating Index, *PHQ-9* Patient Health Questionnaire-9 (depression), *PPT* Pressure pain threshold, *TMJ* Temporomandibular Joint

^*^p < 0.05

### Predictors of TMD

**Fig. 1:**
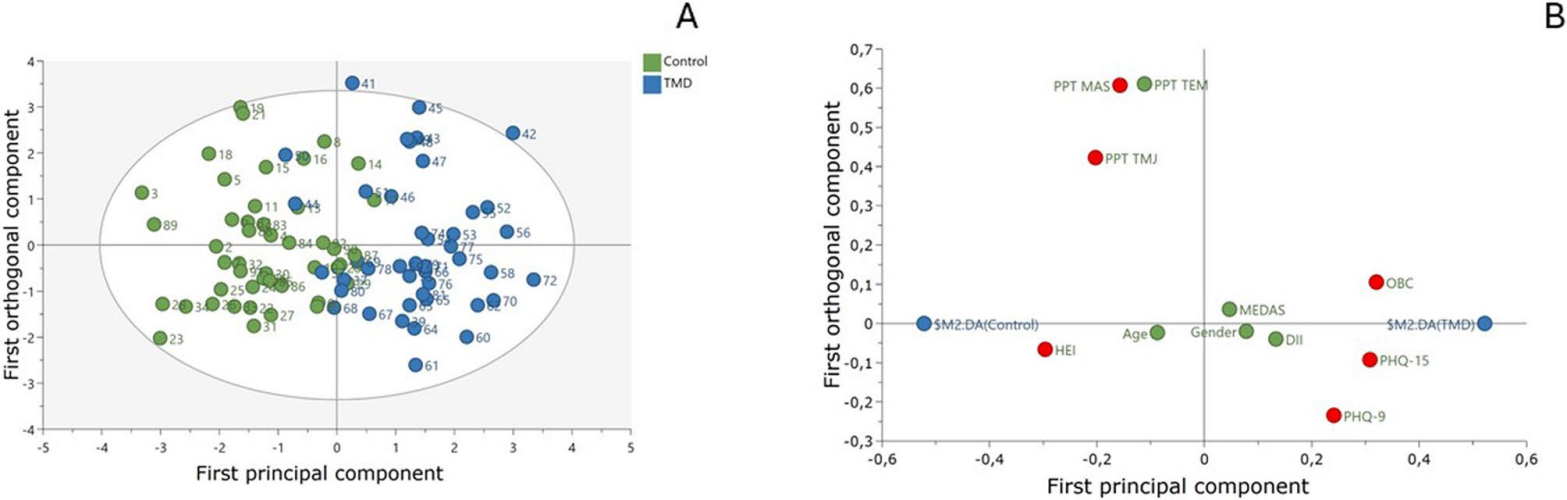
Orthogonal partial least square discriminant analysis (OPLS-DA) of patients with painful temporomandibular disorders (TMD) and healthy control subjects (CONTROL). **A** Score plots showing the separation between each observation in the TMD and CONTROL and **B**) Loading plot showing the variables and their loadings with a VIP-value > 1.0 and p(corr) > 0.4 (red boxes). Green boxes are non-significant variables. Blue boxes represent group (diagnoses) separation. Significant variables correspond to those in Table 3 VIP: Variable Influence on Projection; p(corr): correlation coefficient in multivariate analyses; HEI: Healthy Eating Index; DII: Dietary Inflammatory Index; MEDAS: Mediterranean Diet Adherence Screener; OBC: Oral Behaviour Checklist; PHQ-15: Patient Health Questionnaire-15 (somatic symptoms); HEI: Healthy Eating Index; PHQ-9: Patient Health Questionnaire-9 (depression); PPT: pressure pain threshold, TMJ: temporomandibular Joint, MAS: masseter, TEM: temporalis

The OPLS-DA showed one predictive component that was strongly significant (R2 = 0.644, Q2 = 0.572, CV-ANOVA *p*-value = 8,27e-15) (Fig. 1). The figure clearly shows the separation between patients and controls. There were six variables that differed between the groups, i.e., had a VIP > 1.0 and a p(corr) > 0.4. OBC was the strongest separator, followed by PHQ-15 and HEI. Intercorrelations p(corr) for OBC and PHQ-15 were positive and thus higher in the TMD group. HEI was negative and thus lower in the TMD group (Table 3). As can be seen in the loading plot (Fig. 1) psychosocial variables were more related to the TMD group, whereas PPT and HEI variables were more related to the controls.

**Table 3:** OPLS-DA model of variables discriminating controls from patients with painful TMD

	VIP	*p*(corr)
OBC	1.4	0.8
PHQ-15	1.3	0,7
HEI	1.3	−0.7
PPT-MAS	1.1	−0.4
PHQ-9	1.1	0.6
PPT-TMJ	1.0	−0.5
PPT-TEM	1.0	−0.3
DII	0.5	0.3
Age	0.3	−0.2
Gender	0.3	0.2
MEDAS	0.2	0.1

Six variables discriminated between groups and had a VIP-value > 1.0 and a p(corr) > 0.4. Three variables had a negative p(corr) and were lower in the TMD group, all other variables were higher in the TMD group

*OPLS-DA* Orthogonal Partial Least Square Discriminant Analysis, *VIP* Variable Influence on Projection, *p(corr)* Correlation coefficient in multivariate analyses, *HEI* Healthy Eating Index, *DII* Dietary Inflammatory Index, *MEDAS* Mediterranean Diet Adherence Screener, *OBC* Oral Behavior Checklist, *PHQ-15* Patient Health Questionnaire-15 (somatic symptoms), *HEI* Healthy Eating Index, *PHQ-9* Patient Health Questionnaire-9 (depression), *PPT* Pressure pain threshold, *TMJ* Temporomandibular Joint, *MAS* Masseter, *TEM* Temporalis

## Discussion

The main findings of this study indicate that patients with painful TMDs consume less healthy food and although not statistically significant, exhibit a tendency toward greater intake of pro-inflammatory dietary components than controls. Additionally, TMD pain patients demonstrated higher levels of depressive and somatic symptoms, and maladaptive oral behaviors. Our analysis further revealed that oral behaviors, somatic symptoms, and the consumption of healthy food were the strongest predictive variables associated with painful TMD.

Diet represents an often overlooked yet critical lifestyle factor in the context of chronic pain [36]. Poor dietary habits have been identified as significant risk factors contributing to the development and exacerbation of various chronic pain conditions, including low back pain, migraines and knee osteoarthritis [36–39]. These associations underscore the importance of assessing and targeting dietary habits as a strategic component in chronic pain development and management. In the present study we included participants from two countries known to have a Mediterranean diet, which may partly explain the lack of differences observed in the MEDAS scores between painful TMD patients and controls. Although, adherence to a Mediterranean diet has been linked to numerous health benefits, including anti-inflammatory effects in fibromyalgia and rheumatoid arthritis [40, 41]our findings did not reveal any influence in painful TMDs. It is important to highlight that both assessed groups did not present a good adherence to the Mediterranean diet (MEDAS), since both groups presented a score < 9 [33].

Our data suggested a trend toward higher consumption of pro-inflammatory dietary components among painful TMD patients, although no significant differences were found compared to controls. Chronic musculoskeletal pain, such as TMD pain, is often linked to persistent pro-inflammatory states that promote neuronal structural changes and nociceptive sensitization [42]. In this context, a pro-inflammatory diet (refined sugars, saturated fats and carbohydrates) may contribute to the development or maintenance of painful TMDs symptoms [43–45]. Additionally, independent of nociceptive pathways, diet-induced inflammation in the gastrointestinal system-marked by increased intestinal permeability- may facilitate the systematic passage of cytokines such as TNF-α, IL-1β and IL-6 [46] to the CNS. Then, it could be hypothesized that this systematic inflammatory response could, in turn, contribute to the process of pain and central sensitization in our sample [47]. Future studies are warranted to investigate serum levels of inflammatory biomarkers and their potential association with pain intensity and central sensitization in individuals with painful TMDs. These findings are further reinforced by the lower consumption of vitamin B12 in the painful TMD group, which has been shown to regulate inflammatory mediators in pain models, particularly in COX-mediated second inflammatory phase [48, 49]. Similarly, reduced intake of vitamin C, known for its immuno-supporting and anti-inflammatory effects [50] and garlic which has been associated with a reduction in pro-inflammatory interleukins such as IL-6 [51] may also contributed to a heightened inflammatory state in our TMD sample.

Moreover, participants with painful TMD reported a higher consumption of carbohydrates (high intake of sugar pattern), known to promote pro-inflammatory responses [52, 53]. This dietary pattern likely contributed to the lower HEI scores observed in this group, reflecting poorer diet quality.

Although a higher consumption of carbohydrates is commonly associated with overweight and obesity [47] – conditions linked to chronic musculoskeletal pain disorders such as fibromyalgia [54] and osteoarthritis [55] – overweight was not a characteristic of our sample, despite the increased intake of these components.

Our multivariate analyses demonstrated that lower values of PPT and HEI are significant predictors for painful TMD, which could be interpreted as not eating healthy food may increase pain sensitivity in these patients. A recent study found that a gluten-free diet significantly reduced pain sensitivity in women with myofascial TMD, suggesting dietary modifications could serve as beneficial adjunctive therapy [56]. Moreover, increased pain sensitivity associated with monosodium glutamate ingestion observed in TMD patients highlights the potential for dietary glutamate to exacerbate pain symptoms through peripheral glutamate receptor activation [57, 58]. Taking together, these findings highlight the importance of assessing dietary habits of painful TMD patients. Incorporating tailored dietary interventions into their treatment plans, similarly to what has been implemented in other chronic pain conditions (migraine and fibromyalgia) [59] may contribute to improved outcomes and symptoms management.

Regarding the psychosocial results, previous studies have consistently highlighted psychosocial factors, including somatic symptoms and depression, as significant predictors for the development and chronicity of painful TMDs. Our findings align with existing literature, reinforcing the role of psychosocial variables in TMD pathophysiology. Higher levels of somatic symptoms and maladaptive oral behaviors observed in TMD patients may interact synergistically with dietary habits, particularly through inflammation-driven mechanisms [60, 61].

Furthermore, emerging evidence suggests a link between dietary habits, and psychosomatic symptoms. For instance, dietary patterns low in tryptophan and its metabolism along the kynurenine pathway or diary patterns that alter its metabolism, such as Western-style diets high in saturated fats and sugars, may disrupt serotonin synthesis, potentially exacerbating depressive and somatic symptoms [62, 63]. On the other hand, diets rich in whole, unprocessed foods, such as fruits, vegetables, whole grains, and lean proteins, have been associated with reduced depressive symptoms and somatic complaints [64]. These findings underscore the importance of considering dietary interventions as part of a comprehensive approach to managing TMD and associated psychosomatic symptoms.

### Strengths and limitations

The strengths of this study include the rigorous application of standardized diagnostic criteria (DC/TMD), ensuring robust patient classification. Additionally, the comprehensive assessment of diet, using validated tools like the HEI, DII, and MEDAS, provided a detailed exploration of dietary patterns in relation to TMD. The choice of multivariate analysis as a statistical method further strengthens our results, clearly identifying predictors associated with painful TMD. However, the following limitations need to be acknowledged. One factor is the cross-sectional design that could prevent establishing causality. Another is dietary intake assessment via a single 24-hour recall, which may be subject to recall bias and fails to account for seasonal variation in food consumption, which could limit the accuracy in representing individual’s habitual dietary patterns. Also, our sample size did not allow us to assess the associations between diet and different diagnosis of TMD and was not adjusted to all nutritional variables which could explain the lack of significant differences in DII and MEDAS. Finally, the generalizability of our results may be limited due to the relatively small and geographically restricted sample.

Future research should explore biochemical markers of inflammation to further elucidate the mechanisms linking dietary patterns, psychosocial stressors, and TMD severity [42, 47, 52].

## Conclusion

Taken together, this study highlights dietary quality as a key component of the mechanisms underlying painful TMD. We also found significant associations between psychosocial variables, dietary patterns, pain sensitivity and painful TMD. Notwithstanding, further longitudinal and intervention studies are necessary to clarify causal relationships and optimize dietary recommendations as part of comprehensive TMD management strategies.

## Supplementary Information

Supplementary Material 1. 

## Data Availability

The datasets used and/or analysed during the current study are available from the corresponding author on reasonable request.
