# Correction: Is there a link between diet and painful temporomandibular disorders? A cross-sectional study

**DOI:** 10.1186/s12903-025-07586-8

**Published:** 2026-01-23

**Authors:** Camila Cury Marques, Pedro Miguel Teixeira Carvas Cebola, Idoya Orradre Burusco, Maria García González, Miguel de Pedro, Nikolaos Christidis, Malin Ernberg, Giancarlo De la Torre Canales

**Affiliations:** 1 Egas Moniz Center for Interdisciplinary Research (CiiEM), Egas Moniz School of Health and Science, Almada, Portugal; 2 Orofacial Pain Unit and Sleep Medicine Center, CUF Tejo Hospital, Lisboa, Portugal; 3 Orofacial Pain, Oral Pathology and Dental Sleep Medicine Research Group, Department of Postgraduate Clinical Dentistry, Faculty of Biomedical Sciences, European University of Madrid, Madrid, Spain; 4 Department of Dentistry, Ingá University Center, Uningá, Paraná Brazil; 5 Division of Oral Rehabilitation, Department of Dental Medicine, Karolinska Institutet, Huddinge, Sweden

**Correction: BMC Oral Health 25, 1410 (2025)**

**https://doi.org/10.1186/s12903-025-06835-0**

In this article [1], the author would like to correct the data reported in Table 1 (prevalence of the TMD diagnosis) and the text related to this table in the Results section, that requires adjustment to ensure the accuracy and integrity of the work.

The first paragraph in the Results section should be “The TMD group presented the following frequency of diagnoses: myalgia, 55.5%; arthralgia, 6.6%; disk displacement with reduction, 48.8%; disk displacement without reduction, 2.2% and multiple diagnoses 40%.” instead of “The TMD group presented the following frequency of diagnoses: myalgia, 44.1%; arthralgia, 2.2%; disk displacement with reduction, 48.8%; disk displacement without reduction, 2.2% and multiple diagnoses 37%.”.

Incorrect Table 1: 

**Table 1:** Comparisons of demographic variables between TMD patients and controls (mean ± sd and n (%))

	Groups			
	TMD (*n* = 45)	Controls (*n* = 47)		*p*
Age	40.3 ± 14.4	37.2 ± 10.1		0.24
*Gender*				
Women	37 (82)	32 (68)		0.11
Men	8 (18)	15 (32)		
*Country*				
Portugal	25 (56)	27 (57)		0.21
Spain	20 (44)	20 (43)		
*BMI*	24.1 ± 2.6	24.4 ± 4.6		0.42
*TMD painful diagnosis*				
Myalgia	56 (25)	--		
Arthralgia	2.2 (1)	--		--
DDWR	48.8 (22)	--		
DDWoR	2.2 (1)	--		
Multiple diagnosis	44 (19)	--		

*BMI *Body mass index, *DDWR *Disk displacement with reduction, *DDWoR *Disk displacement without reduction, *TMD *Temporomandibular disorders

*p<0.05

Correct Table 1:

**Table 1:** Comparisons of demographic variables between TMD patients and controls (mean ± sd and n (%))

	Groups			
	TMD (*n* = 45)	Controls (*n* = 47)		*p*
Age	40.3 ± 14.4	37.2 ± 10.1		0.24
*Gender*				
Women	37 (82)	32 (68)		0.11
Men	8 (18)	15 (32)		
*Country*				
Portugal	25 (56)	27 (57)		0.21
Spain	20 (44)	20 (43)		
*BMI*	24.1 ± 2.6	24.4 ± 4.6		0.42
*TMD painful diagnosis*				
Myalgia	25 (55.5)	--		
Arthralgia	3 (6.6)	--		--
DDWR	22 (48.8)	--		
DDWoR	1 (2.2)	--		
Multiple diagnosis	18 (40.0)	--		

*BMI *Body mass index, *DDWR *Disk displacement with reduction, *DDWoR *Disk displacement without reduction, *TMD *Temporomandibular disorders

*p<0.05

## References

[1] Marques CC, Cebola PM, Burusco IO, González MG, de Pedro M, Christidis N, Ernberg M. De La Torre Canales G. Is there a link between diet and painful temporomandibular disorders? A cross-sectional study. BMC Oral Health. 2025;25(1):1410. 40963116 10.1186/s12903-025-06835-0 PMC12442269

