# Supplementary material for: Is there a link between diet and painful temporomandibular disorders? A cross-sectional study
Source: BMC Oral Health. 2025 Sep 17;25:1410. doi: 10.1186/s12903-025-06835-0 (PMC12442269; doi:10.1186/s12903-025-06835-0)
Supplement: Supplementary file 1 — Supplementary Material 1. [file 12903_2025_6835_MOESM1_ESM.docx]

S1. Food intake according to groups

| Element | Control | TMD | | p |
| --- | --- | --- | --- | --- |
| Energy (kcal) | 2338.02 ± 897.78 | | 2344.57 ± 787.3 | 0.970 |
| Protein | 84.84 ± 39.48 | | 82.33 ± 35.41 | 0.749 |
| Total Fat. | 93.67 ± 48.63 | | 101.88 ± 56.69 | 0.457 |
| Sat. Fat. | 34.91 ± 18.29 | | 36.82 ± 16.99 | 0.604 |
| Monounst. Fat. | 26.41 ± 11.68 | | 27.74 ± 13.15 | 0.608 |
| Polyunsat. Fat. | 30.69 ± 13.39 | | 25.5 ± 12.96 | 0.064 |
| Cholesterol | 314.08 ± 140.6 | | 329.4 ± 161.94 | 0.629 |
| Carbohidrates | 206.85 ± 94.33 | | 258.01 ± 84.72 | 0.007* |
| Fibers | 21.71 ± 12.09 | | 19.09 ± 10.32 | 0.266 |
| Iron | 12.55 ± 4.82 | | 11.52 ± 3.44 | 0.239 |
| Magnesium | 230.00 ± 123.23 | | 210.00 ± 120.12 | 0.215 |
| Zinc | 11.50 ± 3.13 | | 10.30 ± 5.52 | 0.093 |
| Vit A | 555.98 ± 313.96 | | 532.61 ± 262.73 | 0.700 |
| Vit C | 255.68 ± 148.89 | | 189.72 ± 132.5 | 0.027* |
| Vit D | 10.56 ± 5.81 | | 8.96 ± 4.85 | 0.158 |
| Vit E | 11.50 ± 4.91 | | 10.30 ± 5.52 | 0.093 |
| Vit B6 | 2.8 ± 1.27 | | 2.36 ± 1.23 | 0.099 |
| Vit B12 | 5.58 ± 2.72 | | 3.95 ± 2.38 | 0.003* |
| Folate | 231.81 ± 131.5 | | 194.67 ± 118.22 | 0.158 |
| Calcium | 760.75 ± 377.16 | | 640.85 ± 414.7 | 0.150 |
| total Sugar | 47.02 ± 27.39 | | 47.58 ± 28.19 | 0.924 |
| Sodium | 2299.58 ± 1347.32 | | 2608.17 ± 1175.49 | 0.244 |
| Coffee | 4.48 ± 2.75 | | 5.57 ± 3.2 | 0.081 |
| Tea | 5.17 ± 3.02 | | 4.29 ± 2.97 | 0.163 |
| Pepper | 5.5 ± 3.13 | | 4.35 ± 2.8 | 0.066 |
| Garlic | 5.33 ± 2.81 | | 3.78 ± 2.63 | 0.007* |
| Curcuma | 5.38 ± 3.05 | | 4.48 ± 2.59 | 0.130 |

*p<0.05
